# Compositional and expression analyses of the glideosome during the *Plasmodium* life cycle reveal an additional myosin light chain required for maximum motility

**DOI:** 10.1074/jbc.M117.802769

**Published:** 2017-09-11

**Authors:** Judith L. Green, Richard J. Wall, Juha Vahokoski, Noor A. Yusuf, Mohd A. Mohd Ridzuan, Rebecca R. Stanway, Jessica Stock, Ellen Knuepfer, Declan Brady, Stephen R. Martin, Steven A. Howell, Isa P. Pires, Robert W. Moon, Justin E. Molloy, Inari Kursula, Rita Tewari, Anthony A. Holder

**Affiliations:** From the ‡ Malaria Parasitology Laboratory,; ** Structural Biology and; ‡‡ Mass Spectrometry Science Technology Platforms, and; ¶¶ Single Molecule Enzymology Laboratory, The Francis Crick Institute, 1 Midland Road, London NW1 1AT, United Kingdom,; the § School of Life Sciences, Queens Medical Centre, University of Nottingham, Nottingham NG7 2UH, United Kingdom,; the ¶ Department of Biomedicine, University of Bergen, Jonas Lies vei 91, 5009 Bergen, Norway,; the ‖ Institute of Cell Biology, University of Bern, Bern, Switzerland, and; the §§ Biocenter Oulu and Faculty of Biochemistry and Molecular Medicine, University of Oulu, Aapistie 7, 90220 Oulu, Finland

**Keywords:** cell motility, invasion, malaria, myosin, plasmodium, glideosome, myosin light chain

## Abstract

Myosin A (MyoA) is a Class XIV myosin implicated in gliding motility and host cell and tissue invasion by malaria parasites. MyoA is part of a membrane-associated protein complex called the glideosome, which is essential for parasite motility and includes the MyoA light chain myosin tail domain–interacting protein (MTIP) and several glideosome-associated proteins (GAPs). However, most studies of MyoA have focused on single stages of the parasite life cycle. We examined MyoA expression throughout the *Plasmodium berghei* life cycle in both mammalian and insect hosts. In extracellular ookinetes, sporozoites, and merozoites, MyoA was located at the parasite periphery. In the sexual stages, zygote formation and initial ookinete differentiation precede MyoA synthesis and deposition, which occurred only in the developing protuberance. In developing intracellular asexual blood stages, MyoA was synthesized in mature schizonts and was located at the periphery of segmenting merozoites, where it remained throughout maturation, merozoite egress, and host cell invasion. Besides the known GAPs in the malaria parasite, the complex included GAP40, an additional myosin light chain designated essential light chain (ELC), and several other candidate components. This ELC bound the MyoA neck region adjacent to the MTIP-binding site, and both myosin light chains co-located to the glideosome. Co-expression of MyoA with its two light chains revealed that the presence of both light chains enhances MyoA-dependent actin motility. In conclusion, we have established a system to study the interplay and function of the three glideosome components, enabling the assessment of inhibitors that target this motor complex to block host cell invasion.

## Introduction

Three stages of the malaria parasite, sporozoites, ookinetes, and merozoites, are invasive to host cells or tissue, and two stages, sporozoites and ookinetes, are motile. Motility and invasion are active processes, and in *Plasmodium* as in other apicomplexan parasites, such as *Toxoplasma gondii*, an actomyosin motor is central to these processes (reviewed in Refs. 1 and 2). Myosin proteins are typically composed of a conserved globular head domain, a neck domain comprising variable numbers of myosin light chain-binding sites, and a diverse tail domain that is often responsible for dimerization and/or cargo binding. The myosin head binds to actin and also contains the motor activity, where ATP hydrolysis is translated into molecular movement (3–6). Myosin A (MyoA),^8^ an Apicomplexa-specific class XIV myosin composed of a head and neck region, but lacking a tail domain, is the motor component of the glideosome protein complex that is attached to the inner membrane complex (IMC), a network of large, flattened vesicles located just below the surface plasma membrane of the parasite (7–9).

Some components of the glideosome have been defined in *T. gondii* and also in *Plasmodium* species. In addition to MyoA, there is a myosin light chain (MLC) that binds to the last few amino acid residues of MyoA, corresponding to part of the myosin neck region and called *T. gondii* MLC1 (10), or myosin tail domain–interacting protein (MTIP) in *Plasmodium* sp. (11). This MLC is involved in anchoring MyoA to the IMC via a palmitoyl modification (12) and thus appears to take on a role usually fulfilled by a myosin tail (*i.e.* anchoring myosin at a specific subcellular location). Recently, two more *T. gondii* MyoA MLCs have been described and named essential light chains 1 and 2 (ELC1 and -2). The terminology follows that used for mammalian muscle myosins with two light chains in which the most proximal is an ELC and the distal is a regulatory light chain (RLC). ELC1 and -2 have been shown to bind to the MyoA neck adjacent to MLC1 (which binds at the RLC site) in a mutually exclusive manner and are important for motor activity (13, 14). Bookwalter *et al.* (15) have demonstrated that a heterologously expressed TgMyoA requires both MLC1 and an ELC in order to move actin at its fastest speed. In addition to MyoA and its associated light chains, a number of glideosome-associated proteins (GAPs) have been described, including GAP45 (16–18) (and its Coccidia-specific homolog GAP70 (19)), GAP50, and a family of three proteins with multiple membrane spans known as GAPMs (20). Another glideosome component, GAP40, has been described in *T. gondii* (19) and has a homologue in *Plasmodium* based on genomic (21) and proteomic studies (22–24), but it has not been shown experimentally to be part of the glideosome in this genus. In *Plasmodium*, most studies on MyoA have been limited to the asexual blood stage parasites in the mammalian host, although its essential role in gliding motility in *P. berghei* ookinetes has been demonstrated in promoter swap experiments (25).

In this study, we have examined the expression and location of MyoA throughout the malaria parasite life cycle, including all invasive stages, and its temporal profile during merozoite and ookinete development. We have examined the composition of the glideosome in asexual blood stages by immunoprecipitation and mass spectrometry of proteins associated with GFP-tagged MyoA, confirming the presence of GAP40 and identifying a new myosin light chain. This essential-type light chain binds to the MyoA neck adjacent to, and in addition to, MTIP. Recombinant PfMyoA binds actin and with both light chains bound demonstrates increased sliding velocity of actin compared with MyoA with just MTIP bound. This is a first important step to reconstituting the *Plasmodium* glideosome *in vitro*, which will be an invaluable system with which to dissect the function of, and interplay between, different components of the complex.

## Results

### Myosin A is a predominantly membrane-associated protein at the periphery of all invasive stages of the P. berghei life cycle

**Figure 1.**
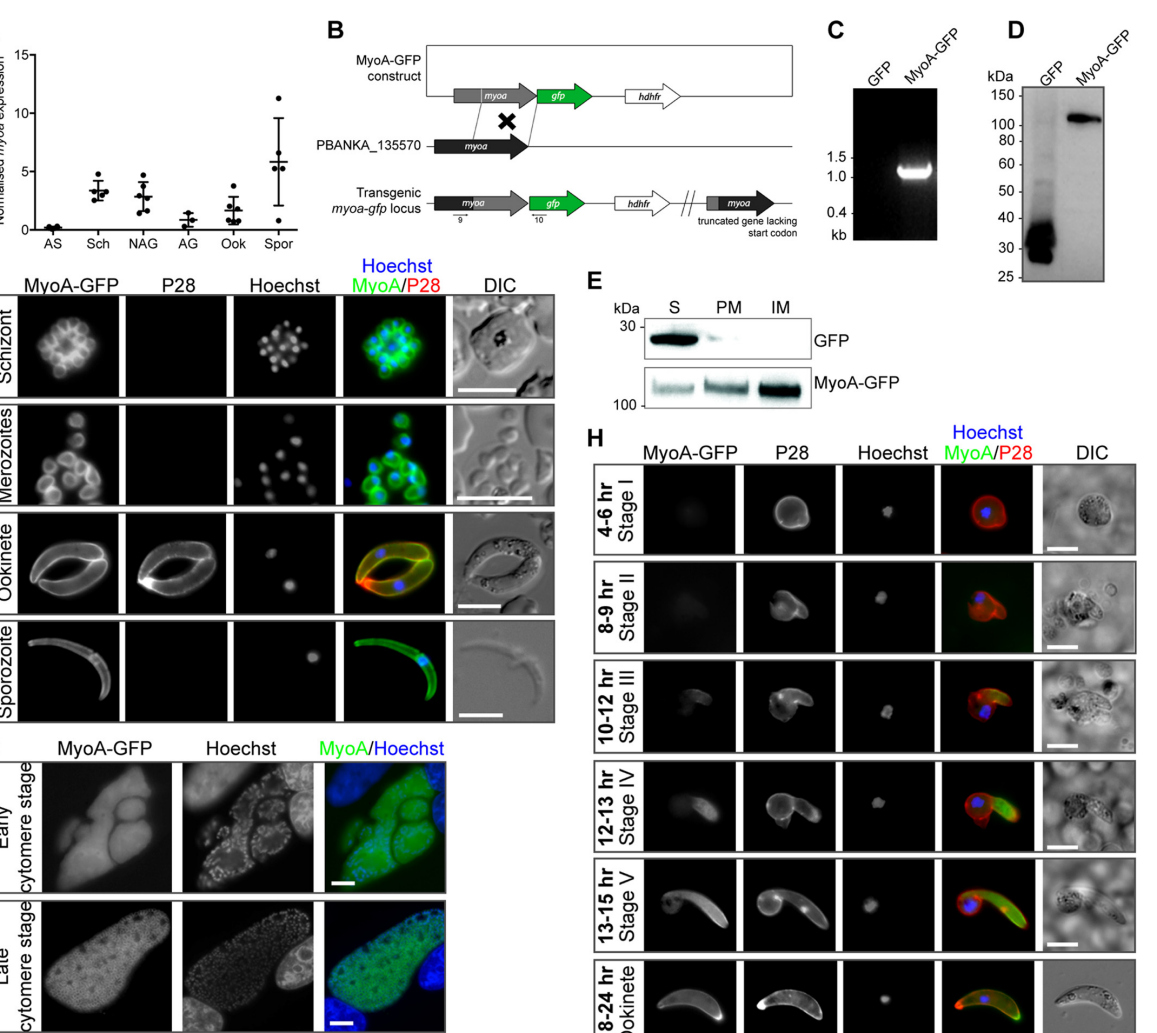
**Expression of GFP-tagged MyoA through the *P. berghei* life cycle.** *A*, transcription of *myoa* analyzed by quantitative RT-PCR normalized against two control genes, *hsp70* and arginine-tRNA synthetase, at different stages of the life cycle. The mean normalized *myoa* expression is shown as a *horizontal bar*, and S.D. values are indicated by *error bars. AS*, asynchronous asexual blood stages; *Sch*, schizonts; *NAG*, non-activated gametocytes; *AG*, activated gametocytes; *Ook*, ookinetes; *Spor*, sporozoites. *B*, a plasmid containing the sequence for the 3′-end of the MyoA coding region fused in frame to the *gfp* sequence, together with a human *dhfr*-selectable marker, was used to insert the *gfp* sequence into the endogenous *myoa* gene. *C*, successful integration into the *P. berghei* genome was confirmed by diagnostic PCR on genomic DNA prepared from wild-type parasites and those with integrated plasmid sequence using primers 9 and 10, which amplify a 1.1-kb DNA fragment from the modified *myoa* locus following correct integration. *D*, expression of the GFP-tagged MyoA was confirmed by Western blotting of lysates from parasites expressing either the unfused GFP or the MyoA-GFP proteins; as indicated, GFP-specific antibodies reacted with either a 29- or a 120-kDa protein, respectively. *E*, extracts of schizonts following hypotonic lysis in the soluble fraction (*S*) and further fractionation of the insoluble material by carbonate buffer into carbonate-soluble (peripheral membrane (*PM*)) and insoluble (integral membrane (*IM*)) fractions. Schizonts from parasites expressing either GFP (*top row*) or MyoA-GFP (*bottom row*) were used. *F*, expression of MyoA-GFP at the invasive and motile stages of the parasite life cycle, detected by live fluorescence microscopy. The parasite nuclei are stained with Hoechst 33342, and the ookinete surface is stained with Cy3-conjugated anti-P28 antibody 13.1. In the *merged color image*, these are *blue* and *red*, respectively, and the MyoA-GFP is *green*. The differential interference-contrast (DIC) bright field images are also shown. *Scale bar*, 5 μm. *G*, expression of MyoA-GFP in liver-stage parasites at the early and late cytomere stages. *Green*, GFP fluorescence; *blue*, Hoechst 33342 staining. *Scale bar*, 10 μm. *H*, temporal profile of MyoA-GFP expression during the six stages of *P. berghei* ookinete development. MyoA-GFP was detected by live fluorescence microscopy associated with the protuberance that grows out of the spherical body only at stage III, eventually forming the motile ookinete. The parasites were co-stained with Hoechst 33342 and with Cy3-conjugated anti-P28 antibody 13.1 as a marker for the zygote and ookinete surface. The merged (MyoA-GFP (*green*), P28 (*red*), and Hoechst (*blue*)) and DIC bright field images are also shown. *Scale bar*, 5 μm.

Expression of the *myoA* gene was detected by quantitative RT-PCR throughout the parasite life cycle, particularly in schizonts and sporozoites but also in non-activated gametocytes (Fig. 1*A*). To examine the synthesis and location of the protein throughout the parasite life cycle, we produced a C-terminal GFP-tagged MyoA expressed from the endogenous genomic locus using a strategy we described recently for *Plasmodium falciparum* MyoA and *Plasmodium berghei* MyoB (26) (Fig. 1*B*). Integration of sequence coding for GFP into the endogenous MyoA locus and expression of the tagged protein was confirmed by PCR amplification from genomic DNA using primers that only produce a product following specific integration (Fig. 1*C*), by Western blotting (Fig. 1*D*), and by fluorescence microscopy (Fig. 1, *F–H*). Tagging MyoA with GFP resulted in no detectable phenotype; there was no effect on the rate of asexual parasite growth, on male gametogenesis (the number of exflagellation centers), the number of oocysts per mosquito gut, or the efficiency of infection via sporozoite inoculation (as judged by the number of days following infected mosquito bite that parasites were observed by microscopy in blood smears). In a subcellular fractionation of schizonts, the protein was largely associated with the peripheral membrane (carbonate-soluble) and membrane (carbonate-insoluble) fractions rather than in the soluble cell lysate (Fig. 1*E*), consistent with its association with the glideosome and the IMC membrane compartments. Using live parasite microscopy, the protein was detected uniformly at the periphery of segmenting schizonts and extracellular merozoites in asexual blood stages and at a similar location in extracellular ookinetes and salivary gland sporozoites (Fig. 1*F*), consistent with location at the IMC. Interestingly, in sporozoites, there was also a clear perinuclear localization. In early cytomere stages of liver-stage schizonts, the signal appeared to be cytosolic, rather than membrane-associated. In late cytomere stages, 55 h after invasion by sporozoites, a clear peripheral location associated with the hepatic merozoites was observed (Fig. 1*G*). The presence of *myoA* mRNA in ookinete stages, as well as in non-activated gametocytes as a translationally repressed transcript, prompted us to examine the temporal profile of protein expression during the 24 h of ookinete development divided into six stages (27) (Fig. 1*H*). MyoA-GFP was not visible in either activated or non-activated gametocytes and was barely detectable in the zygote and stage I and II ookinetes. Following the establishment of morphological polarity at about 10 h after fertilization (stage III), the protein was detectable in the growing protuberance rather than the spherical body in the intermediate retort forms, so-called because of their shape (stages III–V). This location was retained in later stages, with the MyoA-GFP associated with the parasite periphery in fully formed motile ookinetes. There was a concentration of fluorescence detected at the apical tip of the mature ookinete. Immunofluorescence using an antibody against the surface marker P28 was used to visualize the ookinete at all stages of development.

### Expression of MyoA during P. falciparum asexual blood stage development and formation of the glideosome complex

**Figure 2.**
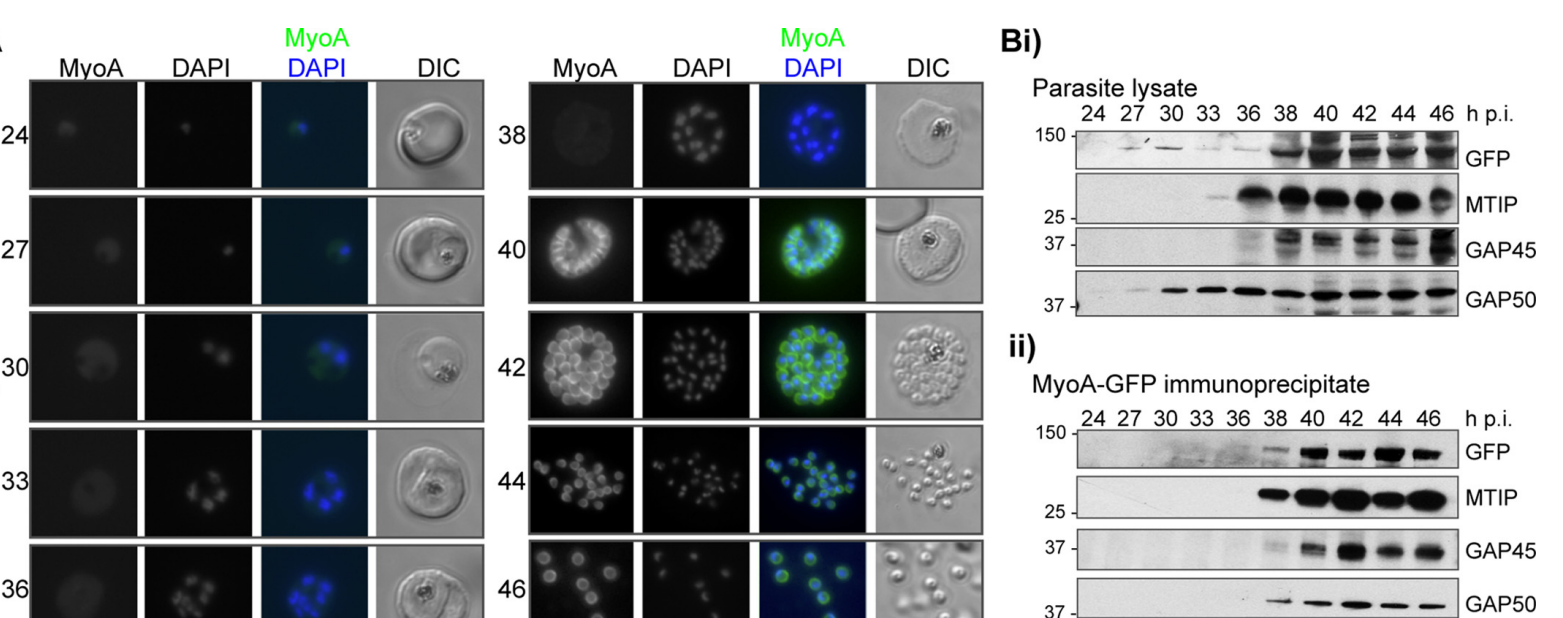
**MyoA-GFP expression and glideosome complex formation during the late stages of intracellular development of *P. falciparum* in the red blood cell.** *A*, MyoA-GFP expression was detected by live fluorescence microscopy, and the nuclei were detected by staining with DAPI. The *merged color image* with MyoA-GFP (*green*) and DAPI (*blue*) and DIC bright field images are also shown. Schizogony starts at around 30 h postinvasion, and MyoA-GFP is detected from 38 to 40 h in multinucleated forms. *Scale bar*, 2 μm. *B*, parasites expressing MyoA-GFP were collected at the indicated time points postinvasion, and lysates were prepared; samples of these lysates (*i*) and of proteins precipitated from them with a GFP-specific antibody (*ii*) were fractionated by SDS-PAGE and probed on Western blots with antibodies to GFP, MTIP, GAP45, and GAP50. Molecular mass markers are indicated in kDa.

The use of *P. falciparum* allows a more detailed analysis of asexual blood stages than can be achieved with *P. berghei.* MyoA-GFP was largely undetectable by live fluorescence microscopy until ∼38 h after merozoite invasion of an erythrocyte but was present in later stages at the periphery of developing segmented schizonts and free merozoites (but not associated with the food vacuole/residual body), consistent with its proposed location at the IMC (Fig. 2*A*). No GFP signal was detected in ring or trophozoite stages, indicating that the protein is not synthesized at this time, and any MyoA carried through with the invading merozoite is degraded rapidly following erythrocyte invasion. Interestingly, the protein was also not detected in early schizonts when the first formation of the IMC can already be detected with, for example, GFP-tagged GAP50 (28) or GAP45 (29) and seen as small ring-shaped structures at the tips of developing merozoites. Western blot analysis of parasite extracts at the corresponding time points (Fig. 2*B* (*i*)) confirmed that GAP50 is present in early schizonts (detectable from 24 h and strongly expressed 30 h postinvasion). Although a small amount of MyoA-GFP can be detected 27–36 h after merozoite invasion, the strongest expression is detected from 38 h postinvasion. A Western blot analysis of the MyoA-GFP immunoprecipitate obtained from these extracts (Fig. 2*B* (*ii*)) indicates that known components of the glideosome complex, MTIP, GAP45, and GAP50, are associated with MyoA from 38 h after erythrocyte invasion by a merozoite. Tagging MyoA with GFP had no effect on the rate of asexual blood stage parasite growth.

### Location of MyoA in P. falciparum during erythrocyte invasion

**Figure 3.**
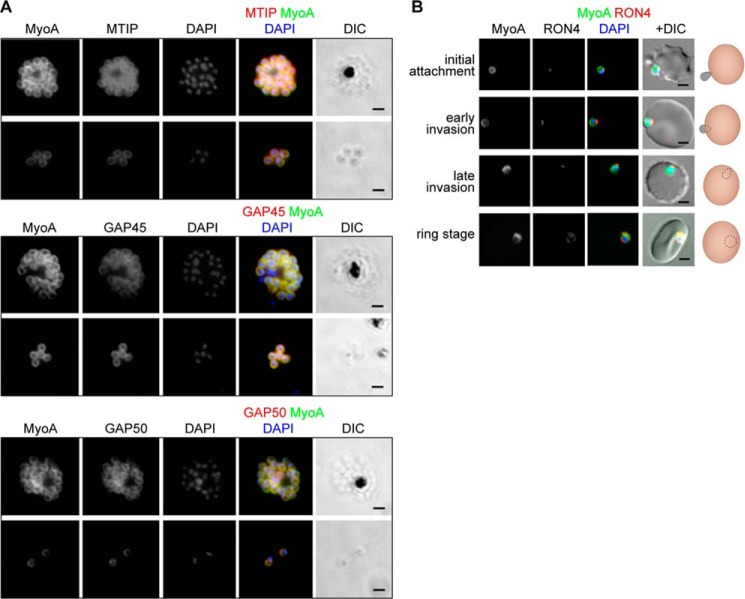
**The location of MyoA during *P. falciparum* merozoite invasion.** *A*, MyoA-GFP is located at the periphery of developing intracellular (*top row* of each pair of images) and free extracellular merozoites (*bottom row*) and is colocalized with antibodies specific for IMC proteins MTIP, GAP45, and GAP50. In the *merged color image*, the MyoA-GFP signal is *green* and antibodies specific for the IMC proteins are *red*; nuclei are stained *blue* with DAPI. The DIC image is also shown. *B*, individual merozoites are captured at different stages of invasion from initial attachment, through early and late invasion to the intracellular ring stage. MyoA-GFP remains peripheral, whereas RON4, initially in the apical rhoptry neck, relocates during invasion. *Merged color images* with MyoA-GFP (*green*), RON4 (*red*), and nuclei (*blue*) and DIC images are also shown, together with a *schematic* of each cell pair. *Scale bar*, 2 μm.

The peripheral location of MyoA-GFP in mature schizonts and free merozoites was confirmed by an indirect immunofluorescence assay (Fig. 3*A*). Antibodies against GFP co-localized with antibodies against MTIP, GAP45, and GAP50, consistent with the presence of these proteins in a complex. During erythrocyte invasion, the peripheral location of MyoA did not change during the transition from initial attachment of the merozoite to the intracellular ring stage at the completion of invasion (Fig. 3*B*). However, thereafter, the signal disappeared until resynthesis during schizogony.

### Identification of GAP40 and discovery of a second MyoA light chain in the P. falciparum glideosome complex

**Figure 4.**
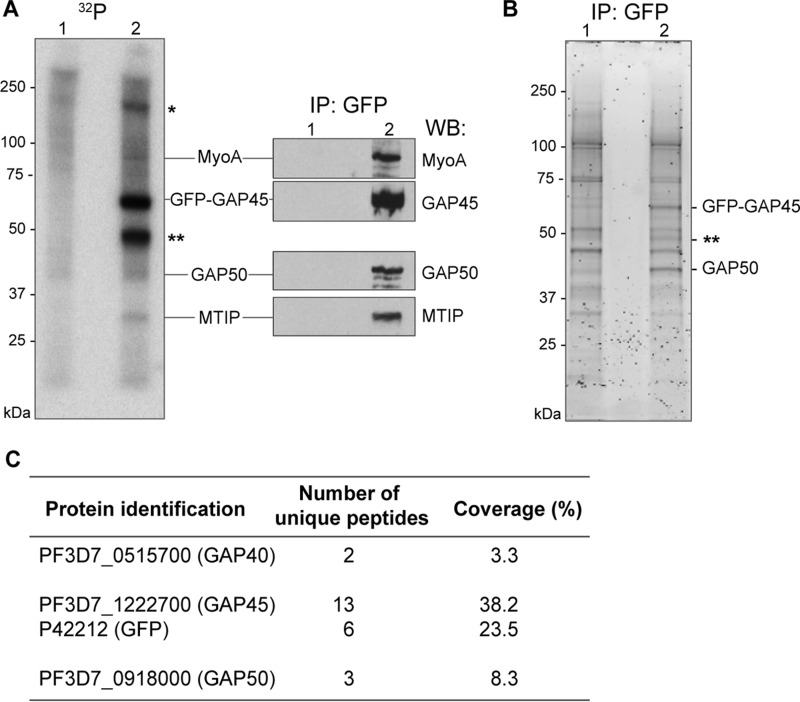
**Identification of GAP40 in the *P. falciparum* glideosome complex.** *A*, wild-type (*lane 1*) or GAP45-GFP–expressing (*lane 2*) *P. falciparum* 3D7 parasites were incubated with radiolabeled phosphate, and proteins binding to a GFP-specific antibody were immunoprecipitated, resolved on an SDS-polyacrylamide gel, and detected by autoradiography. Bands corresponding to two unidentified proteins are labeled with *one* or *two asterisks*. In parallel, the same samples were probed with antibodies to known components of the glideosome complex: MyoA, GAP45, GAP50, and MTIP. *B*, the immunoprecipitation with anti-GFP antibody resin was repeated with unlabeled schizont lysate from wild-type (*lane 1*) or GAP45-GFP–expressing (*lane 2*) *P. falciparum* 3D7 parasites. Immunoprecipitated proteins were fractionated by SDS-PAGE and detected with SYPRO Ruby, and the indicated bands were excised. *C*, proteins identified in the three excised bands by tryptic digestion and mass spectrometry. The number of unique peptides identified for each protein is indicated, along with the percentage of the protein sequence that these peptides cover. *IP*, immunoprecipitation; *WB*, Western blotting.

A number of proteins associated with the glideosome have been identified in both *Plasmodium* and *Toxoplasma* species, and the presence of GAP45, GAP50, and MTIP, some of the known components in *P. falciparum* asexual blood stages, has been confirmed above. To examine what other proteins might be associated, we examined further the protein complex immunoprecipitated with GFP-specific antibodies. To do this, we first used parasites in which GAP45 was tagged internally with GFP (29) and then those in which MyoA was tagged at its C terminus with GFP. In the first instance, we were interested to see whether or not GAP40, which is a highly phosphorylated protein (23, 30–32) present in the *T. gondii* glideosome (19), is present in the *Plasmodium* complex. To visualize phosphorylated proteins associated with GFP-GAP45, we metabolically labeled *P. falciparum* schizont phosphoproteins using [^32^P]phosphate (Fig. 4). As a control, 3D7 schizonts in which GAP45 was unmodified were used (Fig. 4, *lane 1* in *A* and *B*). The glideosome was precipitated from schizont lysates using antibodies to GFP, and ^32^P-labeled phosphoproteins were visualized by autoradiography (Fig. 4*A*). In addition to GFP-GAP45 itself, there were five labeled proteins that were absent from the control samples. The identities of some were confirmed by immunoprecipitation from schizont lysates with GFP antibodies followed by Western blotting with antibodies against known glideosome components MyoA, GAP45, GAP50, and MTIP (Fig. 4*A*). The most prominent of the phosphoproteins, with an apparent molecular mass of just under 50 kDa, was distinct from these four proteins, so its identity was examined further by mass spectroscopy following fractionation of the protein precipitate by SDS-PAGE and tryptic digestion of specific bands (Fig. 4, *B* and *C*). This analysis clearly identified the protein as GAP40 and confirmed the presence of GAP45 and GAP50 in the immunoprecipitate. The high-molecular weight phosphoprotein, marked with an *asterisk* in Fig. 4*A*, was not detected in the SYPRO Ruby-stained immunoprecipitate. High-level phosphorylation of a low-abundance protein is one possible explanation for this discrepancy.

An immunoprecipitate from parasites expressing GFP-tagged MyoA was also analyzed by tryptic digestion, with LC-MS/MS analysis of resultant peptides to identify associated proteins. Again, 3D7 schizonts in which MyoA was unmodified were used as a negative control. Table 1 shows the proteins identified only in the MyoA-GFP precipitate by the presence of two or more specific peptides. This approach confirmed the presence of MTIP, GAP50, GAP45, and GAP40. It also identified GAPM2 and GAPM3, which are two of the three GAPM proteins previously shown to associate with the glideosome and IMC (20). The absence of GAPM1 in the precipitate could be explained by the extremely detergent-resistant property of this protein (20). In addition to these known or expected components of the glideosome, 13 additional proteins were identified; whereas it is likely that some of them represent co-purified contaminants, it cannot be excluded that some represent real components of the complex. For example, it is unlikely that signal peptide peptidase, MESA, and the hexose transporter are part of the glideosome because of their known or predicted subcellular location: the endoplasmic reticulum (33), the parasite-infected erythrocyte surface (34), and the parasite plasma membrane (35), respectively. On the other hand, the protein encoded by PF3D7_1420200 is a tetratricopeptide repeat protein related to TgUNC, demonstrated to be a member of the UCS family of myosin-specific chaperones (15). In addition, of particular interest was the protein encoded by PF3D7_1017500, which is a 134-residue polypeptide with sequence homology to members of the EF-hand superfamily (36) and reminiscent of myosin light chains and other calmodulin-like proteins. This protein is a candidate second light chain for MyoA, particularly because it has been proposed that there is “space” for a second light chain located adjacent to the MTIP-binding site (37), and additional light chains have been characterized for MyoA in *T. gondii* (13, 14). Because of this and data that will be shown below, we subsequently refer to PF3D7_1017500 as PfELC.

**Table 1:** **Identification of proteins immunoprecipitated by GFP-Trap affinity resin together with *P. falciparum* MyoA-GFP using mass spectrometry** All proteins were absent from a control *P. falciparum* immunoprecipitation, where MyoA was unmodified. Proteins are ranked by the percentage of sequence coverage, and previously known components of the glideosome/IMC are in boldface type.

Accession number	Protein description	Molecular mass	Coverage	Peptides
		*kDa*	%	
**PF3D7_1342600**	**Myosin A (MyoA)**	**92.2**	**72.5**	**66**
**PF3D7_1246400**	**Myosin light chain 1, myosin A tail domain–interacting protein (MTIP)**	**23.5**	**31.4**	**7**
**PF3D7_0918000**	**Glideosome-associated protein 50 (GAP50)**	**44.6**	**29.8**	**9**
PF3D7_1017500	Conserved *Plasmodium* protein, unknown function	15.7	24.6	2
PF3D7_1033200	Early transcribed membrane protein 10.2 (ETRAMP 10.2)	38.9	22.8	6
**PF3D7_1222700**	**Glideosome-associated protein 45 (GAP45)**	**23.6**	**19.6**	**4**
PF3D7_0822900	Conserved *Plasmodium* protein, unknown function	138.2	16.2	15
PF3D7_1444300	Putative 1-acyl-*sn*-glycerol-3-phosphate acyltransferase	33.7	12.7	2
**PF3D7_0423500**	**Glideosome-associated protein with multiple membrane spans 2 (GAPM2)**	**42.6**	**12.4**	**2**
PF3D7_1416100	Putative GTP-binding protein, root hair–defective 3 (RHD3) homologue	110.5	12.2	10
PF3D7_1409400	Conserved *Plasmodium* protein, unknown function	30.5	10.7	5
PF3D7_1032500	Putative DER1-like protein (Der1-2)	31.0	10.3	3
PF3D7_1457000	Signal peptide peptidase (SPP)	47.5	8.0	3
**PF3D7_0515700**	**Putative glideosome-associated protein 40 (GAP40)**	**51.8**	**7.5**	**4**
PF3D7_0500800	Mature parasite-infected erythrocyte surface antigen, protein 2 (MESA)	168.2	6.9	6
**PF3D7_1406800**	**Glideosome-associated protein with multiple membrane spans 3 (GAPM3)**	**32.9**	**6.7**	**2**
PF3D7_0912400	Alkaline phosphatase	52.7	5.8	2
PF3D7_0204700	Hexose transporter (HT)	56.4	5.4	2
PF3D7_0824400	Nucleoside transporter 2 (NT2)	67.6	3.8	2
PF3D7_1420200	Tetratricopeptide repeat family protein, putative	147.9	1.9	2

### Bioinformatic and functional analysis of PfELC, a novel P. falciparum MyoA light chain

**Figure 5.**
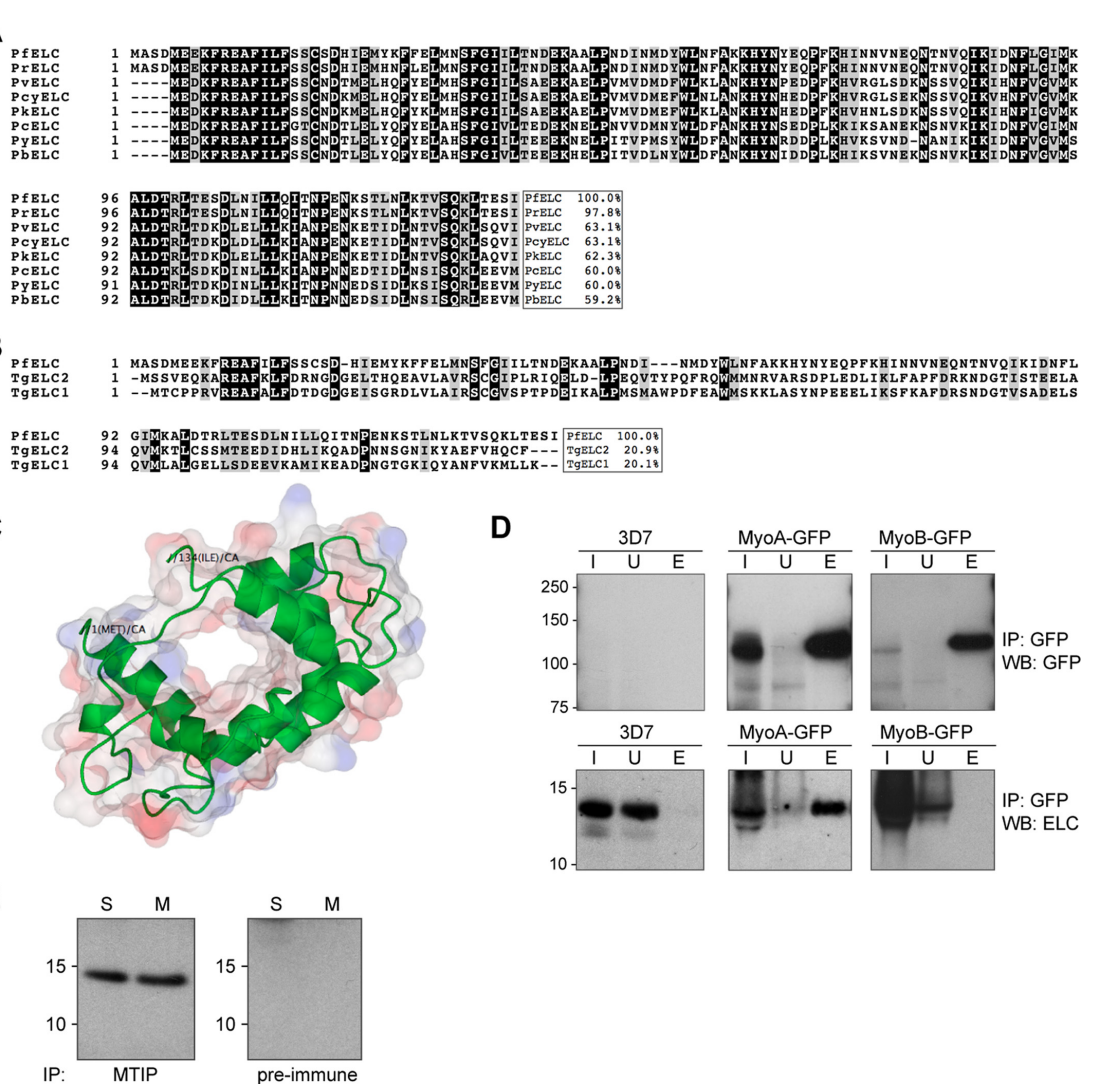
**The putative ELC binds to MyoA but not MyoB and is in the *P. falciparum* glideosome complex together with MTIP.** Shown is alignment of *Plasmodium* ELC homologues (*A*) and PfELC (*B*) with the two *T. gondii* MyoA ELCs. Identical residues are *shaded black*, and biochemically similar residues are *shaded gray*. Percentage identities to PfELC are displayed. Gene identifiers are as follows. *PfELC*, PF3D7_1017500; *PrELC*, *P. reichenowi* PRCDC_1016900; *PvELC*, *Plasmodium vivax* PVX_001745; *PcyELC*, *Plasmodium cynomolgi* PCYB_061180; *PkELC*, *Plasmodium knowlesi* PKNH_0601700; *PcELC*, *Plasmodium chabaudi* PCHAS_0501900; *PyELC*, *Plasmodium yoelii* PY02639; *PbELC*, *P. berghei* PBANKA_0501800; *TgELC1*, *T. gondii* TGME49_069440; *TgELC2*, *T. gondii* TGME49_305050. *C*, structural homology model of ELC generated using Phyre 2. *D*, proteins in extracts from wild-type (3D7) schizonts and those expressing either MyoA-GFP or MyoB-GFP were immunoprecipitated with resin-bound antibodies to GFP and then probed by Western blotting with antibodies to GFP and ELC. *I*, input lysate; *U*, unbound protein; *E*, protein eluted from the resin. *E*, the protein complex containing MTIP also contains ELC. The glideosome complex from lysates of schizonts (*S*) and merozoites (*M*) of 3D7 was precipitated with anti-MTIP antibodies and resolved by SDS-PAGE, and the presence of ELC was determined by Western blotting with specific antibodies. A preimmune antibody sample was used to immunoprecipitate from the lysates as a control. *IP*, immunoprecipitation; *WB*, Western blotting.

The ELC protein is highly conserved across the *Plasmodium* genus, with identities ranging from 59% with *P. berghei* to 98% with *Plasmodium reichenowi* (Fig. 5*A*); however, there is only moderate sequence similarity with the *T. gondii* MyoA ELCs that have been described recently (13, 14). The *Toxoplasma* gene database, ToxoDB, has recently split the gene encoding TgELC1 into 2 genes, TGME49_269438 and TGME49_269442 in the ME49 strain, erroneously in our opinion. Previous releases showed a single gene, TGME49_069440, and it was using this release that Nebl *et al.* (13) were able to assign peptides to TGME49_069440 from immunoprecipitates of the glideosome. Alignment of PfELC and TgELC1 (TGME49_069440) proteins shows 20% identity. Similarly, a second putative essential light chain, TgELC2 (TGME49_305050), that can bind to TgMyoA in place of TgELC1, shows 21% identity with PfELC (Fig. 5*B*). Homology modeling using the Phyre2 structure prediction server (38) suggested considerable structural similarity with calmodulin, which is consistent with PfELC being a functional myosin light chain. The entire sequence was modeled on the structure of the calmodulin moiety of a genetically encoded calcium indicator protein (Protein Data Bank code 3U0K) with 76% confidence (Fig. 5*C*).

### Interactions of PfELC with the glideosome and MyoA

Several approaches were taken to obtain further information on the properties of PfELC. Antibodies were generated to the recombinant protein and used to test for the presence of the protein in immunoprecipitated protein complexes from *P. falciparum* schizont lysates. The immunoprecipitate with GFP-specific antibodies from cells expressing either MyoA-GFP or MyoB-GFP was probed with antibodies raised against recombinant PfELC. This analysis revealed a 14-kDa protein associated with MyoA in the glideosome complex, with very little free unbound protein, and none was detected associated with the MyoB complex (Fig. 5*D*). When the immunoprecipitation was performed with anti-MTIP antibodies and a lysate of schizonts or merozoites from 3D7 parasites, Western blotting with the PfELC-specific antibodies detected a 14-kDa protein in the complex, not only confirming the presence of PfELC but also demonstrating that it is present with MTIP in the same complex (Fig. 5*E*).

**Figure 6.**
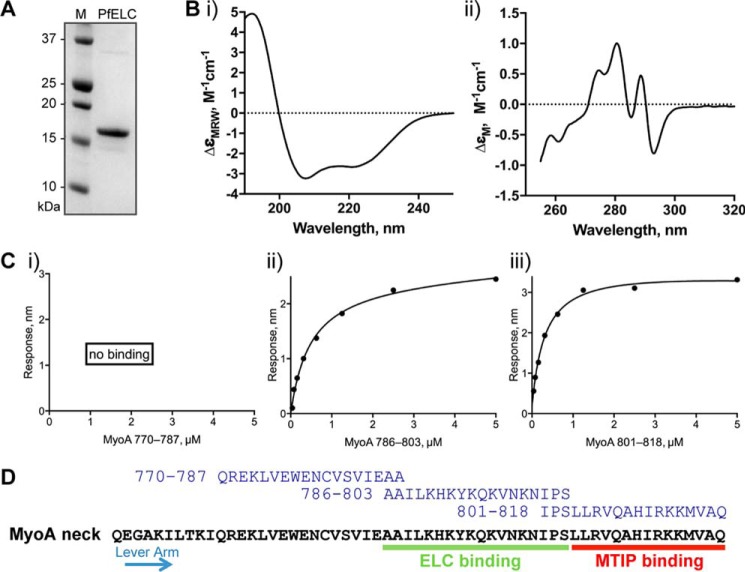
**The putative MyoA ELC binds to the neck region of MyoA.** *A*, purification of recombinant His-PfELC protein. *B*, far-UV (*i*) and near-UV (*ii*) circular dichroism spectra of recombinant protein indicate that it is highly structured, with 32% α-helix, 17% β-sheet, 20% turn. *C*, PfELC binding to immobilized peptides corresponding to residues 770–787, 786–803, and 801–818 of MyoA was measured by biolayer interferometry, using increasing concentrations of PfELC. The dissociation constant (*K_d_*) with S.E. was calculated from each of the curves. *D*, the amino acid sequence of the neck region of MyoA, showing the location of the peptides used for analysis, the MTIP-binding site, and the proposed ELC-binding site.

PfELC was expressed in recombinant form as a His-tagged fusion protein (Fig. 6*A*). Far UV-CD spectra indicated that PfELC is mainly helical, with 32% α helix, 17% β sheet, and 20% turn (Fig. 6, *B* (*i*)). The addition of 1 mm calcium ions had no effect on either the near- or far-UV spectrum, suggesting that calcium does not bind to the protein or that there is no change in secondary or tertiary structure of the protein upon binding. This is supported by the fact that there are no appropriately placed acidic residues that would normally be involved in the coordination of calcium ions in calmodulin-related proteins with canonical EF-hands (39). To examine whether or not PfELC interacts directly with MyoA, the binding of the recombinant protein to peptides derived from the neck region of MyoA was examined. Amino acid residues 760–818 of MyoA constitute its neck region, and MTIP has previously been shown to interact strongly with peptides from the C terminus of the neck (40, 41). Using biolayer interferometry, we measured the affinity of PfELC to overlapping biotinylated peptides corresponding to residues 770–787, 786–803, and 801–818 of MyoA. There was no observable binding to peptide 770–787, but both peptides 786–803 and 801–818 bound to PfELC, with calculated *K_d_* values of 0.48 ± 0.15 and 0.32 ± 0.07 μm, respectively (Fig. 6*C*). There was no effect of 1 mm calcium on the affinity to any of the three peptides. As MTIP has been shown to bind to the more distal MLC-binding site, but not to a peptide representing residues 781–801 of PfMyoA (41), we propose that PfELC binds to a region within 786–803, whereas MTIP binds to a region within 801–818 (Fig. 6*D*).

### The subcellular location of PfELC

**Figure 7.**
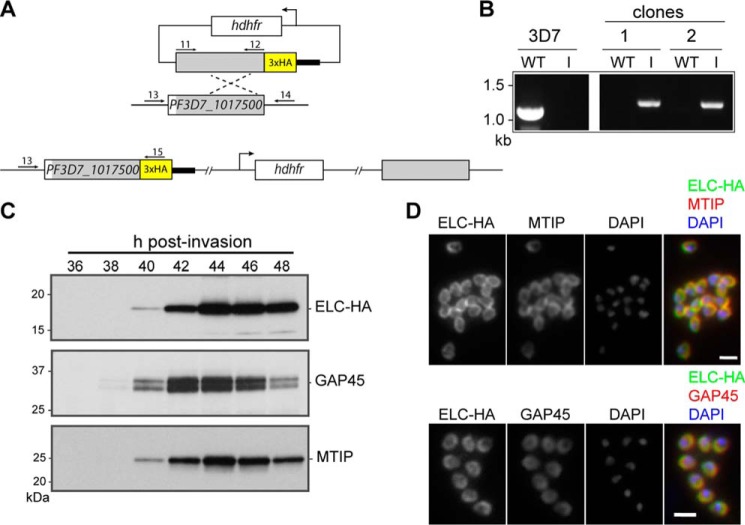
**PfELC expression matches that of known glideosome components in *P. falciparum* schizonts.** *A*, scheme for modifying the PF3D7_1017500 gene locus by single-crossover homologous recombination to C-terminally modify the ELC protein with a triple-HA tag. *B*, PCR screening of parental 3D7 parasites and two independently generated clones. PCR product from the wild-type locus was amplified using primers 13 and 14 (*WT*), whereas a product (*I*) from the modified locus was amplified using primers 13 and 15. *C*, Western blot analysis of HA-tagged ELC, MTIP, and GAP45 proteins present during a time course of schizont development. *D*, immunofluorescent detection of HA-tagged ELC and either MTIP or GAP45 in mature schizonts and merozoites. *Merged color images* are also shown, with ELC-HA (*green*) and MTIP or GAP45 (*red*). Nuclei are stained with DAPI (*blue*). *Scale bar*, 2 μm.

A parasite line expressing an HA-tagged PfELC was generated by single homologous recombination into the endogenous gene locus (Fig. 7*A*), and integration into the *PfELC* locus was confirmed by PCR in two independent clones (Fig. 7*B*). The 17-kDa tagged protein could be detected by Western blot analysis of parasite extracts, and the time of synthesis corresponded to that of MTIP and GAP45 in mature schizonts, peaking at 44–46 h postinvasion (Fig. 7*C*). By 48 h after invasion, many of the schizonts had ruptured, and the merozoites had gone on to invade fresh erythrocytes, explaining the apparent drop in expression of all glideosome components at this time point. Immunofluorescence analysis of fixed blood-stage *P. falciparum* with HA-specific antibodies showed co-localization of the protein with GAP45 and MTIP in late, segmented schizonts (Fig. 7*B*).

### Recombinant PfMyoA binds to actin

**Figure 8.**
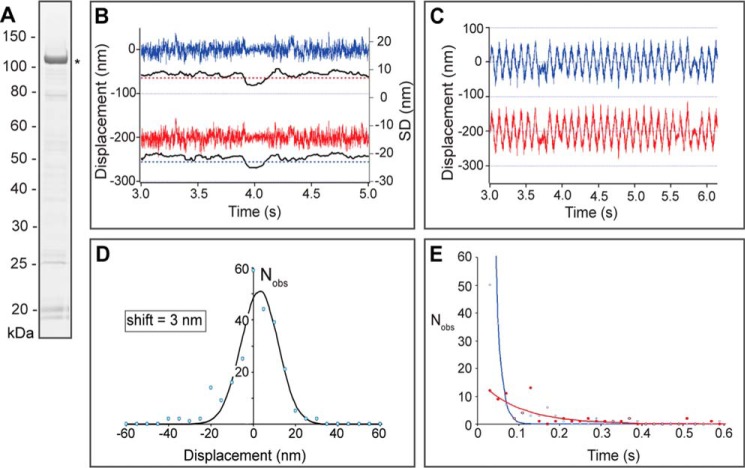
**Recombinant MyoA binds actin.** *A*, Coomassie-stained SDS-PAGE showing affinity-purified GST-MyoA, marked with an *asterisk. B*, an actomyosin event as determined using optical tweezers, with an actin filament held between two beads, a left-hand bead (*blue*) and a right-hand bead (*red*), with the MyoA molecule on a third bead. Because of the high sensitivity required in these single-molecule measurements, Brownian motion causes a significant amount of displacement noise, and when MyoA binds to actin, that event is evident by a sudden decrease in the S.D. of the displacement noise (shown in *black*). Events are scored by thresholding data falling below a fixed value (*dotted red*/*blue line*). Note that movement of the left-hand bead and its S.D. are offset by −200 and −30 nm, respectively, for clarity. *C*, improved visualization of binding events is achieved by moving the beads back and forth using a 10-Hz triangular wave function of 100-nm amplitude. *D*, displacement histogram generated from 244 acto-MyoA–binding events with a fitted Gaussian distribution. The magnitude of the displacement (nm) is displayed on the *x* axis, and the number of observations at that value (*N*_obs_) is displayed on the *y* axis. The average movement caused by the myosin power stroke was measured as 3 nm. *E*, acto-MyoA event lifetime distributions measured at two Mg·ATP concentrations, 8 μm (*blue symbols*) and 0.3 μm (*red symbols*), confirm that the event lifetimes are strongly dependent on [Mg·ATP]. The *x* axis shows the duration of the binding event (in seconds); the *y* axis is the number of observations (*N*_obs_) at that value. The *lines* are single-exponential least-square fits with rate constants 53 s^−1^ (*blue*) and 6.9 s^−1^ (*red*).

As a first step to reconstitute the functional complex *in vitro*, we expressed PfMyoA using an *in vitro* transcription/translation system and examined its ability to bind to actin. Soluble GST-tagged PfMyoA was purified (Fig. 8*A*), and its ability to bind actin was examined using an optical tweezer-based single-molecule system (Fig. 8, *B* and *C*). A total of 244 acto-MyoA–binding events were recorded in 2 μm ATP, a concentration chosen based on the affinity of TgMyoA for mant-ADP (10). The MyoA “working stroke” was 3 nm, as measured from the shift in mean position of the observed event distribution (Fig. 8*D*). Specificity of the acto-MyoA interaction was established using two different ATP concentrations of 0.3 and 8 μm and measuring the duration of acto-MyoA–binding events fitted to a single-exponential decay. The data were consistent with a single rate-limiting step, due to ATP binding to the acto-MyoA rigor complex. At 0.3 and 8 μm ATP, we observed interactions lasting on average 0.1 s (*N*_obs_ = 79) and 0.013 s (*N*_obs_ = 165), respectively, and consistent with Mg·ATP binding and dissociating the acto-MyoA rigor complex with second-order kinetics of ∼15 × 10^6^ m^−1^·s^−1^ (Fig. 8*E*).

### MTIP and PfELC are required for maximum PfMyoA-dependent motility in vitro

**Figure 9.**
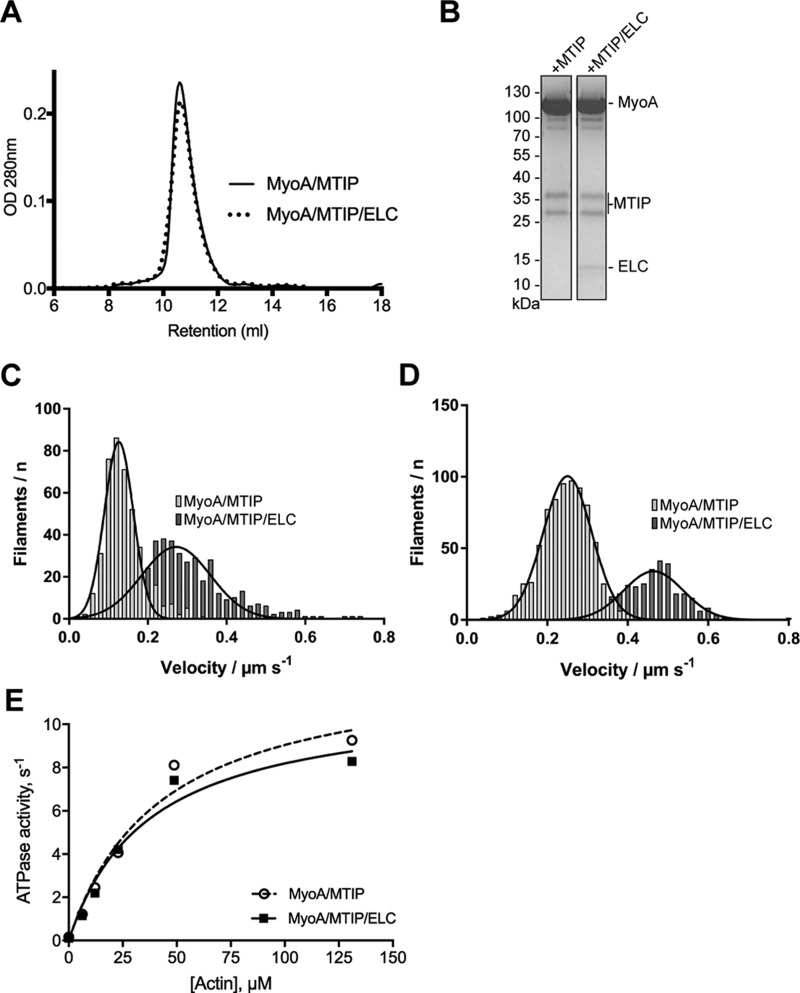
**MTIP/ELC-decorated MyoA shows increased *in vitro* sliding motility.** *A*, size exclusion chromatography of co-expressed MyoA and MTIP (*solid line*) and MyoA, MTIP, and ELC (*dotted line*). *B*, Coomassie-stained SDS-PAGE of the purified MyoA-light chain complexes. *C*, motility of nitrocellulose-captured MyoA complexes; *D*, the corresponding capture of MyoA complexes via an anti-His tag antibody measuring the velocity of actin filament movement in the presence of ATP. A *solid line* represents a Gaussian fit to each corresponding histogram. *E*, the actin-activated ATPase of MyoA-MTIP and MyoA-MTIP-PfELC was estimated using an NADH-coupled assay to quantify the rate of ATP hydrolysis.

To examine the effects of light chain composition on MyoA motility, we expressed and purified recombinant MyoA together with MTIP and MTIP/PfELC. For these studies, we required more material than it was possible to generate using the *in vitro* transcription/translation system that we used in the single-molecule PfMyoA studies described under “Recombinant PfMyoA binds to actin.” We opted for a heterologous expression system that would allow co-expression of PfMyoA and its light chains. Bookwalter *et al.* (15) showed recently that TgMyoA can be expressed well in insect cells but requires Unc45 to fold correctly. Therefore, we followed the same strategy and co-expressed the *P. falciparum* Unc45 homologue and PfHsp90 together with PfMyoA, and we were able to purify functional MyoA in complex with MTIP or with both MTIP and PfELC. Fig. 9*A* shows size-exclusion chromatography traces of the two purifications, indicating a pure, homogeneous complex. Fig. 9*B* shows the complexes separated by SDS-PAGE, indicating that the complexes contain the full complement of light chains included in the co-expression system. A peculiarity is that MTIP migrates as two bands on the SDS-PAGE. We have performed mass spectrometry on each of the two bands to confirm they are both MTIP. We are unsure what is the cause of this aberrant migration. We studied MyoA complexes using an *in vitro* motility assay. MyoA was captured on a coverslip by coating the surface either with nitrocellulose (Fig. 9*C*) or with an anti-His tag antibody (Fig. 9*D*), which orients the head domain away from the surface. Without the addition of ATP, actin filaments bound to the surface and were non-motile. Following ATP addition, the filaments started moving, showing ATP-driven motility (supplemental Movie 1). We also observed significant actin filament fragmentation even at the lowest surface densities used that would still support smooth filament sliding motility (50 μg/ml myosin protein added to the flow-cell surface). In our assay, >95% of actin filaments showed smooth gliding movement with only a small number of non-moving filaments. This observation is firm evidence that our MyoA preparation contained very few so-called “dead-heads” (myosin dead-heads bind actin in an irreversible, tightly bound state). Nitrocellulose- and antibody-captured MyoA-MTIP gliding velocities were 0.13 ± 0.04 μm s^−1^ (*N*_obs_ = 414) and 0.25 ± 0.06 μm s^−1^ (*N*_obs_ = 761), respectively, showing that antibody immobilization increased the gliding velocity ∼2-fold. Furthermore, the MyoA-PfELC-MTIP complex moved filaments ∼2-fold faster than the MyoA-MTIP complex and produced actin gliding velocities of 0.27 ± 0.06 μm s^−1^ (*N*_obs_ = 404) on nitrocellulose and 0.46 ± 0.07 μm s^−1^ (*N*_obs_ = 329) when immobilized via anti-His antibody. The maximum actin-activated ATPase activity (*V*_max_) and actin affinity (*K_m_*) were 12.9 s^−1^ and 42 μm for MyoA-MTIP and 11.2 s^−1^ and 37 μm for MyoA-MTIP-PfELC, respectively (Fig. 9*E*).

## Discussion

We have taken advantage of the ability to GFP-tag MyoA to study in detail its time of synthesis and location in the life cycle of the malaria parasite, using fluorescence microscopy with live parasites expressing the tagged gene from the endogenous genomic locus. No growth phenotype was associated with the tagging of MyoA with GFP. The protein is most abundant in the invasive and motile stages. MyoA is an integral component of the glideosome complex, which is associated with the IMC located below the parasite plasma membrane. In the *P. falciparum* asexual blood stage, there is no IMC in the ring and trophozoite stages, and genesis of the IMC commences soon after the onset of nuclear division, as part of the establishment of polarity for each developing merozoite, and is detected as intracellular ring-shaped structures associated with the individual nuclei (28, 29). At this stage, MyoA-GFP cannot be detected in the cell. However, as the IMC continues to grow, leading eventually to cell segmentation and individual merozoite formation, MyoA accumulates on the IMC, which can be distinguished from the parasite plasma membrane in mature schizonts because it does not surround the food vacuole. In released extracellular merozoites, MyoA-GFP has the characteristic peripheral location but then rapidly disappears following erythrocyte invasion. The mechanism by which the disappearance of the glideosome occurs is undetermined. It is likely that proteasome-mediated degradation of the protein components occurs, although the signaling mechanisms that underlie this are unknown. Throughout the *P. berghei* life cycle, MyoA is most abundant in the extracellular motile and invasive forms (merozoites, ookinetes, and sporozoites). In the sexual stages, although *myoa* mRNA is detected in gametocytes, the protein is barely detectable in gametocytes or the zygote, and protein is only evident once ookinete differentiation is under way. Translational repression is a mechanism of post-transcriptional gene regulation in *Plasmodium*, and *myoa* has previously been shown to be regulated in this way (42, 43), with protein synthesis occurring after fertilization. Following formation of the protuberance that heralds development of the polarized ookinete, MyoA-GFP begins to be deposited at the periphery of the growing protuberance through the retort forms until finally the entire mature motile ookinete is labeled with GFP fluorescence. The deposition of MyoA in this way provides insight into the formation of the IMC in the developing ookinete. It is possible that MyoA activity is important in providing the force required for the developing protuberance to extend away from the spherical body and acquire the characteristic shape of the ookinete. Similarly, the periphery of the salivary gland sporozoite is uniformly labeled with MyoA-GFP. Interestingly, the sporozoite was the only parasite stage in which we observed an additional perinuclear MyoA-GFP localization. It may be that the IMC plays a role in maintaining the shape of the sporozoite. A gene knock-out in *P. berghei* of *PbIMC1a*, which encodes a sporozoite-specific protein with homology to articulins and localizes to the sporozoite IMC, resulted in salivary gland sporozoites with an abnormal morphology. The sporozoites had a characteristic bulge at the position of the nucleus, and the nucleus appeared more rounded than the usual slender nucleus of sporozoites (44). It is possible that MyoA plays a role in maintaining the cellular architecture and shape of the sporozoite in addition to its role in cell motility. The early cytomere stage of liver-stage parasites exhibits cytosolic MyoA-GFP fluorescence, with peripheral localization only detected in the late, segmented cytomere stage. It may be that in the early cytomere stage there is a pool of MyoA that is present before localization to the IMC in the late cytomere stage.

The GFP tag has also proven very useful to purify MyoA and associated proteins in *P. falciparum*. This approach confirmed the presence of MTIP, GAP45, and GAP50, identified previously by Western blotting (26), and GAPM2 and 3, previously identified as components of the apicomplexan glideosome (20). A third member of this family (GAPM1) was not detected here, probably because of its poor solubility in the detergent used. We also identified GAP40 as a component of the glideosome and confirmed that it is one of the major phosphorylated proteins in the complex. Although GAP40 has been described as a component of the *T. gondii* glideosome, it has not previously been shown to be a component of this structure in *Plasmodium*. The fact that we only detected two peptides attributable to GAP40 (3.3% sequence coverage) in our immunoprecipitation/mass spectrometry studies is probably due to the hydrophobic nature of this protein. GAP40 has 10 predicted membrane-spanning regions, with very small loops between them. The two peptides we detected map to the short loop between predicted transmembrane domains 2 and 3. Hydrophobic peptides, such as those that would make up the majority of a GAP40 tryptic digest, are often very poorly ionized in the mass spectrometry workflow, making detection very difficult. This could also explain why GAP40 has, until now, not been detected as a component of the *Plasmodium* glideosome.

In addition to these known or expected components of the *Plasmodium* glideosome, several additional proteins were also identified. Whereas some, such as the signal peptide peptidase or MESA, are likely to be simple contaminants due to their known subcellular location, this cannot be said for some of the other proteins. Their presence or absence in complex with MyoA or other components of the glideosome complex needs to be examined in reciprocal immunoprecipitation experiments using, for example, a variety of GFP-tagged proteins. The tetratricopeptide repeat-containing protein encoded by gene PF3D7_1420200 is similar in sequence to TgUNC in *T. gondii* (TGME49_249480), a member of the UCS family of myosin chaperones (15). Its presence in our immunoprecipitations supports a conserved mechanism of myosin folding in a diverse range of organisms (45). Another of the proteins identified by mass spectrometry, coded by the gene PF3D7_1017500 and annotated as a conserved protein of unknown function, has a sequence suggesting that it might be a myosin light chain. It shows relatively weak homology with *T. gondii* ELCs, indicating that in this case, it would not have been possible to identify the *Plasmodium* ELC by this criterion alone. Interestingly, the abundance of this protein, like that of other glideosome components, including MyoA and MTIP, was significantly decreased in parasites blocked during schizont development (46). Unlike the situation in *T. gondii* tachyzoites, where two ELCs have been identified that bind to TgMyoA in a mutually exclusive manner (14), we found no evidence of light chains other than MTIP and ELC in our immunoprecipitations. This may indicate different modes of regulation and control of the glideosome in these species. Interestingly, there is redundancy in the ELCs of TgMyoA. Either of the genes encoding TgELC1 or -2 can be deleted with no effect on parasite viability; however, deletion of both genes causes a marked reduction in the parasite's ability to invade host cells (14). We have been unable to disrupt the *elc* gene in either *P. berghei* or *P. falciparum*, suggesting that the ELC is essential for parasite viability and that this redundancy does not exist in *Plasmodium* species. This is supported by the inability to disrupt *Pbelc* in the PlasmoGEM project (http://plasmodb.org/plasmo/,^9^ RMgm-1936) (47, 72). Utilizing approaches that are now available in *P. falciparum*, such as the expression of a Cre recombinase that dimerizes in response to the presence of a small molecule to rapidly and inducibly delete genes (48), will be essential to further analyze the specific deficits that occur in parasite growth and development in the absence of PfELC.

We tested the binding of PfELC to several peptides derived from the neck region of MyoA, which encompass the MTIP-binding site at the C terminus of the molecule and the adjacent putative light chain–binding site previously identified by homology modeling (37). PfELC bound to both the known MTIP-binding site and the adjacent proximal site. It has been shown previously that MTIP cannot bind to a peptide composed of residues 781–801 of the MyoA neck (41). This covers all of the sequence of peptide 786–801 used in these studies, which we have shown binds strongly to PfELC. These data, along with the co-precipitation of PfELC with MTIP and MyoA from parasite lysates, confirm that MyoA is decorated with two light chains and that PfELC binds to sequences of MyoA adjacent to the MTIP-binding site. A 30-amino acid region of the *T. gondii* MyoA neck (residues 770–799) has been demonstrated to be the binding site for TgELC1 and -2 (14). The region of PfMyoA that we have demonstrated binds to PfELC is at the C terminus of the equivalent region of PfMyoA, suggesting a similar arrangement of the two light chains on both TgMyoA and PfMyoA. We found no evidence of an effect of Ca^2+^ on the binding of PfELC to MyoA neck peptides or on the structure of PfELC itself, as judged by biolayer interferometry and circular dichroism, respectively. Studies in *T. gondii* have demonstrated Ca^2+^ binding by ELC1, and, by sequence homology, it is assumed that TgELC2 will also bind Ca^2+^. Specific amino acid residues have been suggested (Asp^15^, Asp^17^, and Asp^19^) or demonstrated (Asp^15^) to be required for Ca^2+^ coordination in TgELC1 (14). In the equivalent region of PfELC, these residues are Ser^17^, Cys^19^, and Asp^21^, respectively. The sequence differences here support our observations that Ca^2+^ has no effect on the overall structure of PfELC or on its binding to PfMyoA neck peptides, in that the acidic residues required to coordinate Ca^2+^ in canonical EF-hand motifs (39) are absent. This suggests that there may be subtle differences in the regulation of ELC binding in the *Plasmodium versus Toxoplasma* glideosome.

Additionally, we expressed and purified recombinant MyoA that bound to actin and recombinant MyoA decorated with MTIP or MTIP/ELC that we used to measure sliding velocities by *in vitro* motility assays. The motility was induced by the addition of ATP, and only a small proportion of actin filaments were static, suggesting only a small fraction of non-functional or incorrectly folded myosin heads after a two-step purification. Importantly, MyoA with both MTIP and ELC bound had about a 2 times higher velocity in comparison with MyoA with MTIP alone. This suggests that ELC plays a significant role in *Plasmodium* motility. Our results are in line with results using TgMyoA, which moves actin with approximately 2 times higher velocity when decorated by two light chains (15). It would be interesting to know whether the step size of the MyoA-MTIP-ELC complex is increased compared with that of the MyoA-MTIP complex, as this could be a possible explanation for the increased velocity that we observe in our motility studies for MyoA with its full complement of light chains. Because the actin-activated ATPase activity of MyoA-MTIP and MyoA-MTIP-PfELC was virtually identical, our conclusion is that the increased *in vitro* motility sliding velocity seen with MyoA-MTIP-PfELC compared with MyoA-MTIP alone cannot be explained by a change in catalytic turnover rate (ATPase activity). It is most easily explained by the MTIP/ELC light chain combination having a longer effective lever arm and working-stroke distance, meaning that actin is moved approximately twice as far for each molecule of ATP hydrolyzed.

The glideosome is a molecular motor that has a key role throughout the parasite life cycle. The key components of the glideosome for moving actin filaments are MyoA and its two associated light chains, and we have now established a system where the interplay and function of these three glideosome components can be studied. This will also allow the development and assessment of inhibitors that target the glideosome function and block invasion of host cells.

## Experimental procedures

### Ethics statement for animal work

Animal work at the University of Nottingham was approved by the local animal welfare and ethical review body and by the United Kingdom Home Office under project license numbers 40/3344 and 30/3248 in accordance with the United Kingdom Animals (Scientific Procedures) Act 1986 and in compliance with European Directive 86/609/EEC for the protection of animals used for experimental purposes. 6–8-week-old female Tuck-Ordinary (TO) outbred mice (Harlan) were used for these experiments.

At the University of Bern, experiments were conducted in strict accordance with the guidelines of the Swiss Tierschutzgesetz (animal rights laws) and approved by local authorities. The BALB/c mice used in these experiments were between 6 and 10 weeks of age and bred in the central animal facility of the University of Bern.

### Analysis of PbMyoA gene expression by quantitative RT-PCR

Blood stage *P. berghei* parasites from infected mice (day 4 postinfection) were cultured *in vitro* (49), and the following day, schizont-infected cells were purified on a 60% (v/v) Nycodenz/PBS gradient. Gametocytes were purified on a 48% (v/v) Nycodenz gradient and washed twice in RPMI 1640 medium, and gamete formation was activated by transfer to ookinete medium (RPMI 1640 containing 25 mm HEPES, 25% fetal bovine serum, 10 mm sodium bicarbonate, 50 mm xanthurenic acid at pH 7.6) for 30 min at 20 °C. For ookinete preparation, parasites from mice day 5 postinfection were placed in ookinete medium for 24 h at 20 °C, other parasite stages were lysed in red blood cell lysis buffer for 30 min, and then ookinetes were purified on a 63% (v/v) Nycodenz gradient in PBS. On day 14 post-feeding, mosquito guts were dissected and crushed in a loosely fitting homogenizer to release sporozoites, which were then quantified using a hemocytometer (50).

Total RNA isolation, cDNA synthesis, and PCR analysis were as described previously (49). Expression was determined using the Pfaffl method (51), using *hsp70* (PBANKA_0818900) and arginyl-tRNA synthetase (PBANKA_1434200) as reference genes. All primer sequences are available on request.

### Parasite genetic modification

#### 

##### GFP tagging of P. berghei genes

*P. berghei myoa* (PBANKA_1355700) was modified by single homologous recombination to insert a sequence coding for a C-terminal GFP tag (Fig. 1*B*), using the p277 vector that contains a human *dhfr* selection cassette (52). An 833-bp region of *Pbmyoa* starting 1.86 kb downstream of the ATG start codon and omitting the stop codon was amplified using primers 7 and 8 and inserted into the vector using KpnI and ApaI restriction sites. The construct was linearized by digestion with PacI and used to transfect the *P. berghei* ANKA 2.34 line by electroporation (53). The electroporated parasites were injected into naive mice, and transgenic parasites were selected with pyrimethamine (49) and analyzed by diagnostic PCR using the primers 9 and 10 to determine correct integration of the *gfp* sequence at the targeted locus (Fig. 1*C*). Western blotting with GFP-specific antibodies was used to confirm expression of full-length tagged protein of the predicted size (Fig. 1*D*).

##### Tagging of P. falciparum genes

*P. falciparum* (3D7) was cultured in RPMI 1640 medium containing Albumax II according to previously described methods (54). GFP tagging of *P. falciparum* MyoB, MyoA, and GAP45 has been described previously (26, 29). For HA-tagging of *P. falciparum* ELC at its C terminus, a 632-bp DNA fragment of the gene was amplified using primers 11 and 12 and cloned via XmaI and AvrII sites into the pHH4-HA vector,^10^ a derivative of pHH1 (55). 100 μg of DNA was used to transfect ring-stage *P. falciparum* as described previously (56). Transgenic lines were selected with 10 nm WR99210 (a gift from Jacobus Pharmaceuticals). Plasmid integration was monitored by diagnostic PCR screening with primers 13 and 14 to detect the unmodified locus and primer pair 13 and 15 to detect integration (Fig. 7*B*).

### Antibodies

Polyclonal rat anti-PfMyoA antibodies were raised to a peptide of the sequence MVNKINELNNYFRINSTFINKSENE by Pepceuticals Ltd. according to their standard protocol. Mouse anti-PfELC antibodies were raised by immunization of female BALB/c mice with recombinant His-tagged protein (production of which is described below). Other primary polyclonal antibodies and mAbs used were anti-PfMTIP (40), anti-PfGAP45 (18), anti-PfGAP50 (26), anti-PfRON4 mAb (57), and anti-PbP28 mAb 13.1 (58), which are described in the referenced works. Rabbit anti-GFP^11^ and rat anti-HA (monoclonal 3F10; Roche Applied Science) antibodies were used to detect tagged proteins. Species-specific Alexa Fluor 488– and 594–conjugated secondary antibodies were obtained from Molecular Probes.

### Microscopy

#### 

##### Live imaging of GFP-tagged parasite lines

*P. berghei* asexual blood stage infections in mice were initiated by intraperitoneal injection of parasite-infected blood. The asexual, gametocyte, and ookinete stages were observed using Hoechst 33342 dye (Molecular Probes) in ookinete medium and Cy3-conjugated mouse mAb 13.1 to recognize the p28 protein on the surface of activated female gametes, zygotes, and ookinetes. To examine mid-gut infection, guts were dissected 14 days after mosquito feeding on infected blood and mounted under Vaseline-rimmed coverslips after staining with Hoechst 33342 for 10–15 min. On day 21 post-feeding, guts and salivary glands were dissected and crushed separately in a loosely fitting homogenizer to release sporozoites, which were then quantified using a hemocytometer and used for imaging. Microscopy was performed using a Zeiss AxioImager M2 microscope (Carl Zeiss, Inc.) fitted with an AxioCam MRm digital camera. *P. berghei* liver stage parasites were prepared and imaged as described previously (26), with the exception that infections were performed in HeLa cells.

*P. falciparum*-infected red blood cells were prepared for live imaging as described previously (26, 29) and viewed using an Axio Imager M1 microscope (Carl Zeiss).

##### Indirect immunofluorescence assay

Thin smears of parasites were fixed with 4% paraformaldehyde for 30 min at room temperature. The fixed cells were permeabilized with PBS containing 0.1% Triton X-100 for 5 min followed by blocking in 3% BSA (w/v) in PBS at 4 °C overnight. Primary antibodies were diluted as required; species-specific Alexa Fluor 488– and 594–conjugated secondary antibodies (Life Technologies) were used to visualize primary antibody binding. All antibody dilutions were carried out with 3% BSA (w/v) in PBS. Slides were mounted for microscopic examination using Prolong Gold anti-fade reagent with DAPI (Life Technologies). Slides were viewed using a Zeiss Axioplan 2 microscope, and images were captured using a Zeiss Axiocam MRc digital camera and Axiovision version 4.8.2 software and prepared for publication using Adobe Photoshop.

To image stages of invasion of erythrocytes, tightly synchronized PfMyoA-GFP–expressing late-stage schizonts were mixed with erythrocytes and samples taken after 2, 5, 8, 10, and 30 min and fixed in solution with 4% paraformaldehyde with 0.01% glutaraldehyde for 60 min at room temperature. Further processing was carried out as described previously (59).

### Preparation of parasite lysates, immunoprecipitation of GFP fusion proteins, and analysis of the precipitates by Western blotting and LC-MS/MS

#### 

##### Parasite lysates

Schizonts from MyoA-GFP, GAP45-GFP, and 3D7 *P. falciparum* parasite lines were first lysed in 0.15% (w/v) saponin in PBS and harvested by centrifugation, and then proteins were extracted using 10 cell pellet volumes of ice-cold lysis buffer (0.5% Nonidet P-40, 150 mm NaCl, 10 mm Tris-HCl, pH 7.5, 1× Complete protease inhibitors (Roche Applied Science)). Samples were cleared by centrifugation at 100,000 × *g* for 10 min. The detergent concentration in the samples was reduced to 0.2% by dilution with 150 mm NaCl, 10 mm Tris, pH 7.5, 1× Complete protease inhibitors.

For *P. berghei*, subcellular fractionation was performed as described previously (60). Briefly, cell pellets of MyoA-GFP or WT-GFP schizonts were resuspended in hypotonic lysis buffer (10 mm Tris-HCl, pH 8.4, containing 5 mm EDTA and protease inhibitors), freeze/thawed twice, and then incubated for 1 h at 4 °C. Following centrifugation at 100,000 × *g* for 30 min, supernatant was collected as the soluble protein fraction. The remaining pellet was resuspended in carbonate solution (0.1 m Na_2_CO_3_, pH 11.0, containing protease inhibitors), incubated for 30 min at 4 °C, and then centrifuged at 100,000 × *g* for 30 min. The resulting supernatant was classified as the peripheral membrane fraction, and the pellet was washed and solubilized in 4% SDS and 0.5% Triton X-100 in PBS and classified as the integral membrane fraction. Equal amounts of these three fractions were analyzed by Western blotting using anti-GFP antibody.

##### Radiolabeling P. falciparum phosphoproteins

*P. falciparum* schizonts (39 h postinvasion) were enriched by centrifugation through 70% Percoll (GE Healthcare). The purified schizonts were washed twice with phosphate-free RPMI 1640 medium containing 10% human serum and incubated at 37 °C for 30 min. Then 25 MBq of [^32^P]phosphate (PerkinElmer Life Sciences) was added to the culture, and the incubation was continued for 2 h. The schizonts were then washed twice with serum-free RPMI 1640 medium. The [^32^P]phosphate-labeled schizonts were used for immunoprecipitation using the GFP-Trap system (Chromotek) as described below.

##### Immunoprecipitation

Parasite lysates were precleared by incubation with blocked agarose beads for 1 h, and then the supernatant was incubated with GFP-Trap agarose beads for 2 h at 4 °C with end-over-end rotation. The beads were washed extensively with 150 mm NaCl, 10 mm Tris, pH 7.5, and then subjected to a further wash in 300 mm NaCl, 10 mm Tris, pH 7.5. Finally, the beads were resuspended in 5 volumes of 2× SDS-PAGE sample buffer and boiled for 5 min before fractionation of the proteins by SDS-PAGE.

##### Western blotting

Cell lysates were separated under reducing conditions on NuPAGE gels (Life Technologies). Proteins were transferred to nitrocellulose and blocked in 5% (w/v) nonfat milk in PBS containing 0.2% Tween 20. Primary antibodies were diluted in 5% (w/v) nonfat milk in PBS containing 0.2% Tween 20. Species-specific horseradish peroxidase-conjugated secondary antibodies (Bio-Rad) were used, and the signal was developed using enhanced chemiluminescence (GE Healthcare). Blots were exposed to Biomax MR film (Eastman Kodak Co.), and images were prepared for publication using Adobe Photoshop.

##### Protein identification by LC-MS/MS

Proteins immunoprecipitated from GFP-GAP45 parasite lysates were run on a 10% NuPAGE BisTris gel (Life Technologies) and stained with SYPRO Ruby (Thermo Scientific). The protein bands were visualized using a Pharos FX Plus molecular imager and excised using an EXQuest spot cutter (both from Bio-Rad). Proteins immunoprecipitated from MyoA-GFP or 3D7 parasite lysates were run 4 mm into a 10% NuPAGE BisTris gel (Life Technologies) and then excised using a clean scalpel blade. In both cases, proteins were reduced for 1 h in a solution of 20 mm DTT, 200 mm ammonium bicarbonate, 50% acetonitrile (ACN) and alkylated for 20 min in a solution of 5 mm iodoacetamide, 200 mm ammonium bicarbonate, 50% ACN, before overnight trypsin digestion. The resulting digests were analyzed by LC-MS/MS using an Ultimate 3000 nanoRSLC HPLC, equipped with a 50 cm × 75-μm Acclaim Pepmap C18 column, coupled to an LTQ Orbitrap Velos Pro equipped with a Nanoflex electrospray source (all from Thermo Scientific). A gradient of 6–32% ACN/0.1% formic acid over 48 min was used at a flow rate of 0.3 μl/min. The Orbitrap was operated in data-dependent acquisition mode with a survey scan at 60,000 resolution and up to the 10 most intense ions selected for MS/MS. Raw files were processed using Proteome Discoverer version 1.3 (Thermo Scientific) with Mascot version 2.4 (Matrix Science) as the search engine against the appropriate protein database. A decoy database of reversed sequences was used to filter the results at a false detection rate of 1%.

### Bioinformatic analyses

Alignment of ELC sequences was performed using CLC Sequence Viewer version 6 (Qiagen), and graphics were prepared using BoxShade. A structural model of PfELC was constructed using the Phyre 2 protein fold recognition server (38). Images were prepared using CCP4 molecular graphics software (61).

### Cloning and expression of recombinant PfELC

RNA was prepared from late *P. falciparum* parasites (∼36 h postinvasion) using TRIzol (Life Technologies) and cDNA made from this using the reverse transcription system with oligo(dT) primers (Promega). Primers 16 and 17 were used to amplify *elc* from cDNA, and the product was cloned into the pET46-Ek-LIC expression vector (Novagen) using ligation-independent cloning. After verification by sequencing, the plasmid was transformed into BL21 (DE3) cells (Stratagene), and protein expression was induced by the addition of isopropyl β-d-1-thiogalactopyranoside to 1 mm for 3 h at 37 °C. The cell pellet was lysed using Bugbuster (Novagen) with 25 units of Benzonase/ml and 1× complete protease inhibitors without EDTA (Roche Applied Science). The lysate was cleared by centrifugation at 20,000 × *g* and was applied to a column of nickel-nitrilotriacetic acid-agarose (Qiagen), followed by extensive washing of the column with 20 mm imidazole in phosphate buffer (GE Healthcare). The protein was eluted in 3 column volumes of phosphate buffer with 0.5 m imidazole. The eluate containing the His-ELC protein was collected and concentrated using a Vivaspin 20 concentrator with a 5-kDa molecular mass cut-off (Sartorius). Buffer exchange into 50 mm NaCl, 10 mm Tris-HCl, pH 8.0, was carried out using a PD10 column (GE Healthcare).

### Biophysical analysis of PfELC and its interaction with MyoA neck peptides

#### 

##### Peptides

Overlapping peptides representing amino acids Gln^770^–Ala^787^, Ala^786^–Ser^803^, and Ile^801^–Gln^818^ of the PfMyoA neck were synthesized by Mimotopes with biotin at the N terminus. Each peptide had an SGSG linker between the biotin and 18 amino acids of the MyoA neck. The C termini of the peptides were capped with an amide to remove unnatural charge, apart from peptide 801–818, which was left as a free acid.

##### Far-UV CD

Far-UV CD spectra were recorded on a Jasco J-815 spectropolarimeter fitted with a cell holder thermostatted by a CDF-426S Peltier unit. CD measurements were made at a protein concentration of 10 μm PfELC in 25 mm Tris-HCl, pH 8.0, 50 mm NaCl using fused silica cuvettes with a 1-mm path length (Hellma). All spectra were recorded with 0.1 nm resolution and baseline-corrected by subtraction of the buffer spectrum. Secondary structure content was estimated using methods described by Sreerama and Woody (62).

##### Biolayer interferometry

*P. falciparum* ELC binding to peptides was analyzed by biolayer interferometry using the Octet Red system (ForteBio) at 25 °C in 96-well microplates, as described previously (63). All assays were performed in 100 mm Tris-HCl, pH 8.0, containing 0.005% (v/v) Tween 20, according to the manufacturer's instructions. A baseline was established over 4 min, and biotinylated peptides (0.4 μg/ml) were loaded onto streptavidin sensors for 10 min. The sensors were washed for 4 min and then incubated with PfELC at different concentrations (0.04–5 μm) for 800 s. The data were analyzed to calculate a dissociation constant (*K_d_*) using non-linear regression with a one-site binding model and a least-squares fit (GraphPad Prism version 6).

### In vitro expression and purification of recombinant MyoA

MyoA was expressed in a wheat germ cell-free protein synthesis system (ENDEXT® Technology, CellFree Sciences) (64). Recombinant plasmid DNA using recodonized template DNA was amplified using specific primers and cloned into the vector pEU-E01G-TEV-N1 to express N-terminal GST-tagged protein. For the *in vitro* transcription reaction, 2 μg of DNA was added to a transcription premix (11.5 μl of nuclease-free water, 1× transcription buffer, 2.5 mm NTP mix, 1 unit/μl RNase inhibitor, 1 unit/μl SP6 RNA polymerase) in a total volume of 20 μl and incubated at 37 °C for 6 h, according to the manufacturer's instructions. The quality of mRNA produced was monitored by gel electrophoresis. The bilayer reaction system (CellFree Sciences) was used to translate mRNA into protein. mRNA (10 μl) was added to 10 μl of wheat germ extract (WEPRO® 1240) supplemented with 40 ng/μl creatine kinase and carefully transferred directly below the SUB-AMIX solution (0.3 mm of each l-amino acid, 100 mm potassium acetate, 2.7 mm magnesium acetate, 16 mm disodium creatine phosphate, 0.4 mm spermidine, 1.2 mm ATP, 0.25 mm GTP, 4 mm dithiothreitol, and 30 mm HEPES-KOH (pH 7.8)) and incubated for 20 h at 15–16 °C. Soluble GST-tagged MyoA was purified using glutathione-Sepharose beads (GE Healthcare); supernatant was incubated with the resin for 2 h at 4 °C, followed by extensive washing with 20 mm Tris-HCl, pH 8.0, 250 mm NaCl, 0.5 mm TCEP. The beads were resuspended in 3 volumes of 20 mm Tris-HCl, pH 8.0, 100 mm NaCl, 10 mm reduced glutathione to elute the GST-tagged protein. This was followed by buffer exchange into PBS on a PD10 column (GE Healthcare).

### Single-molecule optical tweezer studies to examine the MyoA interaction with actin

The optical tweezer assay, developed to measure the force and movement produced by individual myosin molecules interacting with an actin filament, was used in the well-established three-bead format with the experimental conditions as described (65–67). The experimental apparatus is based around a Zeiss inverted microscope, and experiments were performed in a flow-cell chamber created using a microscope slide and coverslip. The solution buffers and other conditions were identical to those used in earlier studies (67). A single, rhodamine-phalloidin labeled, actin filament, comprising 1:5 biotin-labeled actin to plain actin, was captured between two neutravidin-coated beads (∼1-μm diameter) using the dual-beam optical tweezers. A third, surface-immobilized bead that was sparsely coated with MyoA was then positioned in close proximity to the actin filament. Interactions between the surface-bound MyoA and the actin filament were recorded by monitoring movement of the beads held in the optical tweezers using two 4-quadrant, photodiode detectors. To facilitate visualization, the beads were moved backward and forward using a 100-nm amplitude, 10-Hz triangular wave. Individual events were then automatically detected by measuring changes in amplitude of the imposed triangular oscillation, and event amplitudes were histogrammed. Data were acquired and saved to computer memory using custom software, with a sampling rate of 10 kHz, and later analyzed using IgorPro software (WaveMetrics Inc., Lake Oswego, OR). Individual acto-MyoA force-producing events were identified as described previously (65), and the MyoA power-stroke amplitude and kinetics were analyzed by histogram analysis of the data.

### Expression in insect cells and purification of recombinant MyoA with light chains

Following the example of Bookwalter *et al.* (15), we co-expressed Pf*Hsp90* and *Unc45* chaperones together with full-length MyoA, PfELC, and MTIP in Sf21 cells. By sequence homology, PF3D7_1420200 is the *P. falciparum* orthologue of *Toxoplasma* Unc45 (http://plasmodb.org/plasmo/).^9^ Codon-optimized genes were designed and synthesized (Life Technologies) for insect cell expression. A codon-optimized *Hsp90* (PF3D7_0708400) was a kind gift from Dr. Will Stanley. *PfUnc45* was cloned under the polyhedrin promoter in the pFL vector (68) and *Hsp90* under the control of the p10 promoter in the same vector. *MyoA* (PF3D7_1342600) was cloned into the pFL vector and fused with N-terminal StrepTagII and *gfp* under the polyhedrin promoter. *MTIP* (PF3D7_1246400) and *ELC* (PF3D7_1017500) genes were cloned into the pFL vectors under the control of the polyhedrin promoter with N-terminal hexahistidine tags. Each construct was transformed in MAX Efficiency DH10Bac cells (Invitrogen), bacmids were isolated, and the gene integrations were confirmed using standard protocols (Bac-to-Bac, Invitrogen). Sf21 cells (Gibco) were transfected using FuGene 6 (Promega) as described (69). After two rounds of virus amplification, Sf21 cells were co-infected with untitered recombinant viruses in ratios of 0.75:1:1:1 for MyoA/Unc45/MTIP/ELC or 0.75:1:1 for MyoA/Unc45/MTIP, and cells were harvested 3 days postinfection. We used 1.5 ml of MyoA-expressing virus per 1 × 10^8^ cells, and for the others we used 2 ml per 1 × 10^8^ cells. Cells were collected, washed with PBS, resuspended in the lysis buffer (20 mm Hepes, pH 7.5, 5% trehalose dihydrate (w/v), 1 mm ATP, 0.5 mm TCEP, 2.5 mm MgCl_2_, 0.5 mg of avidin, 1× SigmaFast EDTA-free protease inhibitor), sonicated, and clarified at 38,000 × *g*. Remaining supernatant was applied into two interconnected 1-ml Strep-Tactin superflow columns (IBA GmbH), washed with lysis buffer, washing buffer 1 (20 mm Hepes, pH 7.5, 5% trehalose dihydrate (w/v), 150 mm KCl, 0.5 mm TCEP), and washing buffer 2 (20 mm Hepes, pH 7.5, 5% trehalose dihydrate (w/v), 0.5 mm TCEP). MyoA complexes were eluted with 20 mm Hepes, pH 7.5, 5% trehalose dihydrate (w/v), 0.5 mm TCEP, and 3.5 mm d-desthiobiotin. MyoA light chain–containing fractions were pooled, concentrated, and run over a Superdex 200 increase 10/300GL column (GE Healthcare) in washing buffer 2. After a size-exclusion step, fractions containing MyoA-light chain complexes were pooled, concentrated, snap-frozen in liquid N_2_, and stored at <−76 °C until use.

### In vitro motility assays

Motility assays were essentially performed as in Butt *et al.* (70). Our assay buffer, AB (25 mm imidazole-HCl, 25 mm KCl, 1 mm EGTA, 4 mm MgCl_2_, pH 7.4) was repeatedly degassed and flushed with argon and stored in a hypodermic syringe to prevent oxygen contamination. An oxygen scavenger, COG (20 mm DTT, 0.2 mg/ml glucose oxidase, 0.5 mg/ml catalase, 3 mg/ml glucose, 0.5 mg/ml BSA) was added to buffers used during fluorescence imaging and stored at room temperature during experiments. Microscope flow-cell chambers were constructed using double-sided tape (Tesa), and flow-cell volume was ∼8–12 μl. To capture MyoA-light chain complexes by anti-His antibody, nitrocellulose-coated coverslips were incubated with neutravidin (Thermo Fisher Scientific) for 4 min. The flow chamber was then washed four times with buffer AB and then blocked (twice) with AB/BSA (AB with 0.5 mg/ml BSA) for 2 min. Biotinylated anti-His antibody (Qiagen) was diluted 1:5 in AB/BSA and applied into the flow cell for 4 min. The non-bound antibodies were removed by washing twice with AB/BSA, and MyoA complexes were applied for 2 min, before washing the flow cell twice with AB/BSA and applying rhodamine-phalloidin–labeled F-actin in AB/COG for 2 min. We used MyoA-MTIP and MyoA-MTIP-PfELC at concentrations of 0.25 and 0.27 mg/ml for nitrocellulose capture and 0.11 and 0.05 mg/ml for anti-His tag capture, respectively. The flow cell was washed twice with AB/COG and then viewed by fluorescence microscopy. Assays were performed using an Axioskop 40 fluorescence microscope with a Zeiss PlanNeofluar ×100, 1.3 numeric aperture objective lens. Fluorescence was excited by a mercury arc lamp using a rhodamine filter set (excitation filter HQ535/50, dichroic mirror Q565LP, and emission filter HQ605/75; Chroma Technology), and light emitted from the rhodamine-phalloidin–labeled actin filament specimen was imaged onto an image-intensified charge-coupled device camera (IC-310, Photon Technology International), where, at the magnification used, each pixel corresponded to 100 nm at the object plane. Sequences of video frames were captured every 40 ms using a frame grabber card (Multipix Imaging Ltd.). The laboratory was air-conditioned, and the experimental temperature was kept at 23 °C. Filament specimens were tracked automatically with GMimPro (71) using non-default settings Q-threshold 3 and track length 5 after saving each image sequence as a median of five consecutive image blocks. Non-motile or short tracks were removed manually. From the resulting average velocities, frequency distribution histograms were calculated (a bin size of 0.02), and a Gaussian curve was fitted with GraphPad Prism version 7.

### Actin-activated ATPase assay

The actin-activated ATPase of MyoA-MTIP and MyoA-MTIP-PfELC was estimated using an NADH-coupled assay to quantify the rate of ATP hydrolysis. The assay buffer contained 160 units/ml lactate dehydrogenase, 400 units/ml pyruvate kinase, 1 mm phospho(enol) pyruvate, 0.5 mm ATP, and 0.3 mm NADH in the motility assay buffer, AB. The myosins (MyoA-MTIP or MyoA-MTIP-PfELC) were added to a final concentration of 0.13 μm, and phalloidin-stabilized filamentous actin was added over the range of 7–130 μm. The rate of ATP hydrolysis was calculated from the rate of change in NADH absorbance measured in a temperature-controlled spectrophotometer at 340 nm. Experiments were conducted at 23 °C. The data were fit to the Michaelis–Menten equation using non-linear regression with a least-squares fit in GraphPad Prism.

### Primers

Sequences of primers used in these studies are available on request.

## Author contributions

J. L. G. assisted with conception of the project, experimental design, interpretation of results, preparation of figures, and writing of the manuscript as well as the design, execution, and analysis of experiments on the novel ELC. R. J. W. constructed the MyoA-GFP *P. berghei* parasite and analyzed MyoA expression throughout the asexual blood and insect stages of the life cycle. J. V. performed experiments with insect cell expressed recombinant MyoA and assisted with the writing of the manuscript. N. A. Y. constructed the MyoA-GFP *P. falciparum* parasite and analyzed MyoA-GFP expression and interaction with other glideosome partners. M. A. M. R. designed and executed the experiments to identify the expression of GAP40 in the glideosome. R. R. S. analyzed the expression of MyoA-GFP in the hepatic stage of the parasite life cycle. J. S. analyzed the expression of MyoA at all stages of the *P. berghei* life cycle and was responsible for preparation of these figures. E. K. designed plasmids to genetically modify *P. falciparum*, developed the methods to examine erythrocyte invasion, and contributed to the analysis of these data. D. B. assisted with all of the work to produce genetically modified *P. berghei* and their maintenance and analysis. S. R. M. was responsible for the design and analysis of the data from all the biophysical experiments with ELC. S. A. H. performed and interpreted all of the mass spectrometry analysis of proteins and their complexes. I. P. P. prepared the MTIP and ELC baculovirus constructs and viruses. R. W. M. assisted with the experiments to generate tagged MyoA in *P. falciparum* and the analysis and interpretation of the expression by microscopy. J. E. M. and I. K. analyzed and interpreted the work involving recombinant MyoA and assisted with the writing of the manuscript. R. T. organized, coordinated, analyzed, and interpreted all of the work involving the use of *P. berghei* and assisted with the writing of the manuscript. A. A. H. conceived and coordinated the project, analyzed and interpreted the data, and organized and wrote large sections of the manuscript. All authors read and approved the final manuscript.

## Supplementary Material

Supplemental Data

## Footnotes

9 Please note that the JBC is not responsible for the long-term archiving and maintenance of this site or any other third party hosted site.

10 E. Knuepfer, unpublished data.

11 E. Knuepfer and A. A. Holder, unpublished data.

## Footnotes

8 The abbreviations used are:

MyoA: myosin A
ACN: acetonitrile
ELC: essential light chain
GAP: glideosome-associated protein
IMC: inner membrane complex
MTIP: myosin tail domain interacting protein
TCEP: tris(2-carboxyethyl)phosphine
mant-ADP: 2′-/3′-*O*-(*N*′-methylanthraniloyl)-ADP
MLC: myosin light chain
RLC: regulatory light chain
BisTris: 2-[bis(2-hydroxyethyl)amino]-2-(hydroxymethyl)propane-1,3-diol
DIC: differential interference-contrast.
