# Supplementary material for: Compositional and expression analyses of the glideosome during the Plasmodium life cycle reveal an additional myosin light chain required for maximum motility
Source: J Biol Chem. 2017 Sep 11;292(43):17857–75. doi: 10.1074/jbc.M117.802769 (PMC5663884; doi:10.1074/jbc.M117.802769)
Supplement: Supplemental Data [file supp_292_43_17857__index.html]

Compositional and expression analyses of the glideosome during the Plasmodium life cycle reveal an additional myosin light chain required for maximum motility — Compositional and expression analyses of the glideosome during the Plasmodium life cycle reveal an additional myosin light chain required for maximum motility — The glideosome needs two light chains for maximum motility — Supplemental Data 

# Compositional and expression analyses of the glideosome during the *Plasmodium* life cycle reveal an additional myosin light chain required for maximum motility

## Supplemental Data

- motility movie (.avi, 8.6 MB) - Movement of actin filaments by myosin complexes
